# Cognitive Function and Proteomic Changes in Patients with Autoantibody‐Positive Neurodegenerative Dementia

**DOI:** 10.1002/alz70857_096919

**Published:** 2025-12-24

**Authors:** Cuibai Wei

**Affiliations:** ^1^ Xuanwu Hospital, Capital Medical University, Beijing, Beijing, China; Innovation Center for Neurological Disorders and Department of Neurology, Xuanwu Hospital, Capital Medical University, Beijing, Beijing, China

## Abstract

**Background:**

Neurodegenerative dementia (ND) is characterized by progressive cognitive decline. The role of autoantibodies in ND remains controversial, as they may contribute to neurological damage or, under certain pathophysiological conditions, exert protective effects. In this study, we aimed to analyze the clinical characteristics of patients with autoantibody‐positive ND and investigate potential molecular mechanisms using proteomics.

**Method:**

The study included 13 patients with autoantibody‐positive ND, 13 with autoantibody‐negative ND, and 13 cognitively normal controls. Demographic characteristics, laboratory test results, and neuropsychological scores were compared across groups. Differentially expressed proteins (DEPs) were identified through proteomic analysis. Bioinformatics approaches were employed to explore potential pathways and mechanisms. Multiple linear regression analysis was performed to examine the effects of protein levels on cognitive function and their interaction with autoantibody status.

**Result:**

The autoantibody‐positive and autoantibody‐negative ND groups displayed similar overall cognitive status, levels of depression or anxiety symptoms, and mental and behavioral abnormalities. Performance across specific cognitive domains was also comparable. However, the autoantibody‐positive ND group demonstrated better daily living abilities and enhanced frontal lobe cognitive function compared to the autoantibody‐negative ND group. Proteomic analysis identified 162 DEPs associated with autoantibodies. These proteins were enriched in pathways related to endocytosis, endoplasmic reticulum protein processing, tight junctions, and cellular adhesion. Further analysis revealed that ARRB1, CAPN2, PPP2R1A, and UGGT1 exhibited inconsistent effects on Frontal Assessment Battery (FAB) and Alzheimer's Disease Cooperative Study‐Activities of Daily Living (ADCS‐ADL) scores in the ND+ and ND‐ groups.

**Conclusion:**

This study offers a novel exploration of the clinical and biological mechanisms underlying autoantibody‐positive neurodegenerative dementia. It also identifies proteins that may influence neuropsychological characteristics, potentially paving the way for personalized treatment strategies.

